# Idiopathic Granulomatous Mastitis Presenting as Bilateral Breast Abscesses

**DOI:** 10.5334/jbsr.2565

**Published:** 2021-12-21

**Authors:** Jesper Dierickx, Sofie Dekeyzer, Filip Vanhoenacker

**Affiliations:** 1 AZ Sint-Maarten Mechelen and University of Ghent, BE; 2 AZ Sint-Maarten Mechelen, BE; 3 AZ Sint-Maarten and University (Hospital) Antwerp/Ghent, BE

**Keywords:** granulomatous mastitis, MRI, ultrasound, mammography

## Abstract

**Teaching point:** Idiopathic granulomatous mastitis is a rare disease that may present as breast abscesses and may mimic other inflammatory, infectious, or neoplastic disorders on imaging.

## Case Presentation

A 43-year-old woman presented with erythema and swelling of the areola and the peri-areolar area of both breasts. She did not have any relevant clinical history.

**Figure 1:**
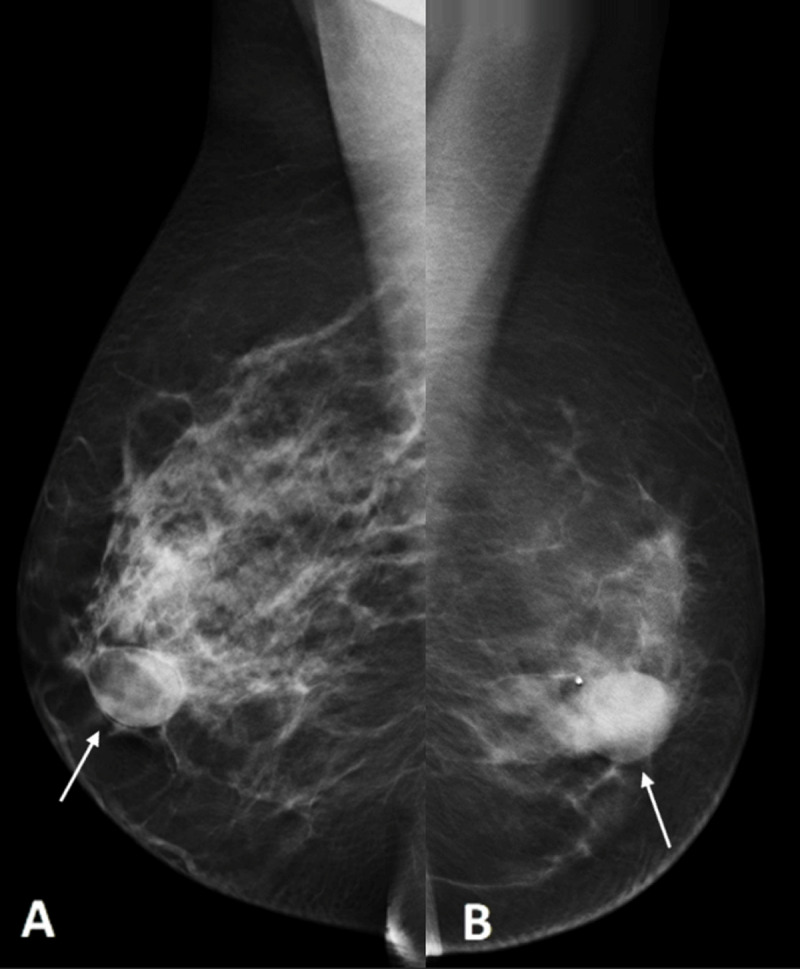


**Figure 2:**
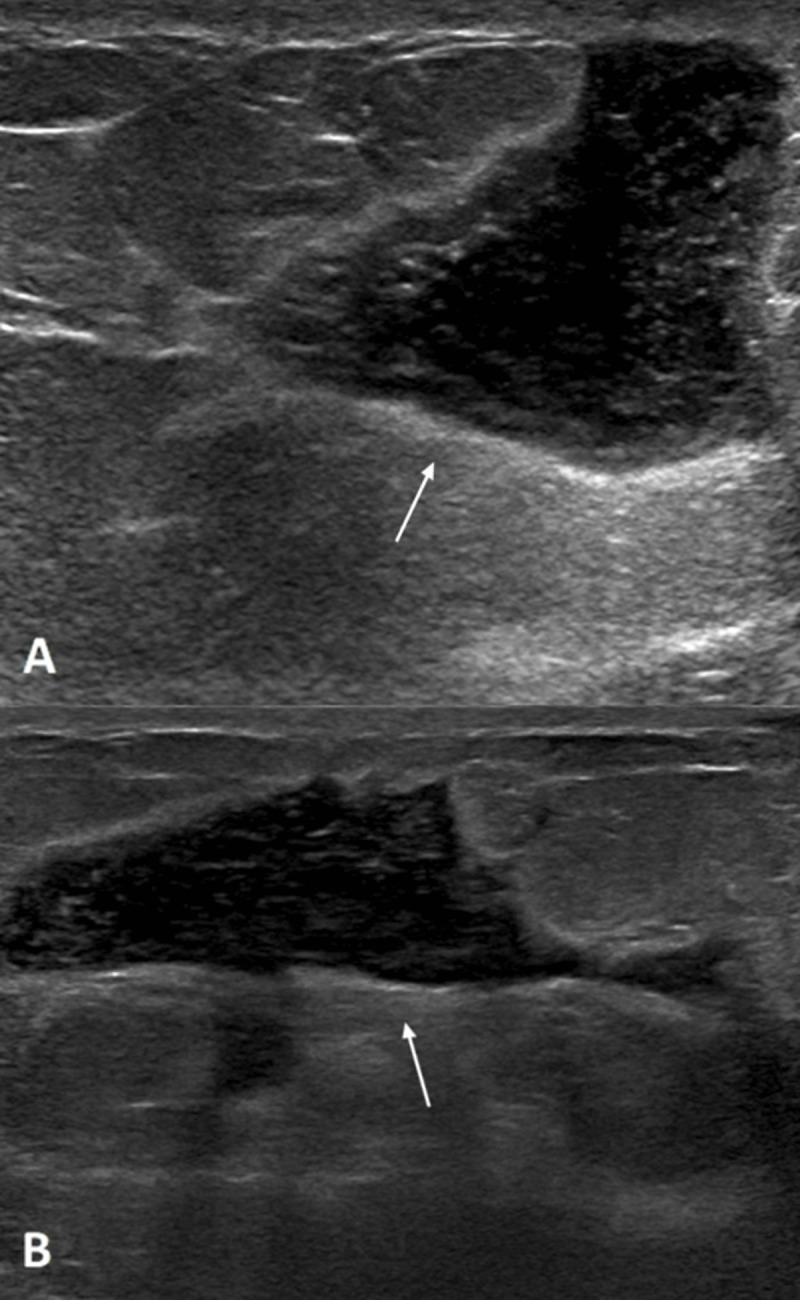


Digital breast tomosynthesis demonstrated well-delineated retroareolar lesions in both breasts, with central lucency and a dense periphery in the right breast and a radiodense lesion in the left breast (***Figure 1A*** and ***1B***, white arrows). Ultrasound confirmed bilateral well-delineated, heterogeneous hypoechoic collections with posterior acoustic enhancement in the retroareolar area (***Figure 2A*** right breast, ***Figure 2B*** left breast, white arrows).

**Figure 3:**
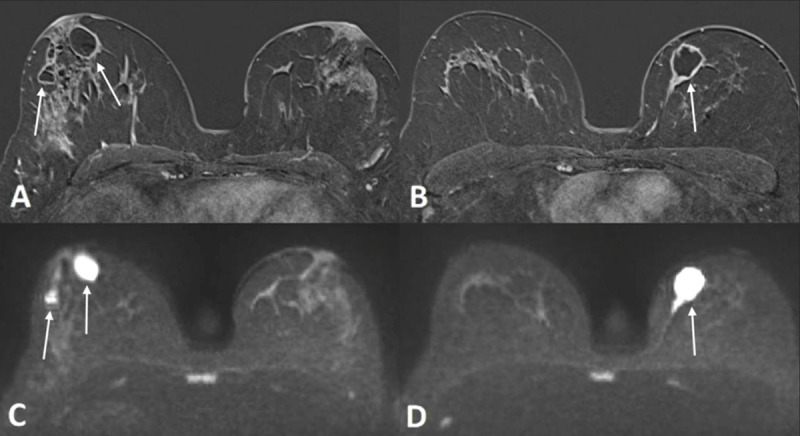


Magnetic resonance imaging (MRI) demonstrated round and oval masses with smooth margins and peripheral enhancement in both breasts on contrast-enhanced subtraction images (***Figure 3A*** and ***3B***, white arrows). Diffusion restriction was observed in all lesions (DWI; ***Figure 3C*** and ***3D***, white arrows). The overlying skin and areola were thickened.

Histopathologic examination of biopsy specimens of both breasts revealed idiopathic granulomatous mastitis with abscesses formation.

## Comment

Granulomatous mastitis (GM) is a rare chronic benign inflammatory breast disease. There are two subtypes. Specific granulomatous mastitis (SGM) is associated with systemic granulomatous disease, including sarcoidosis and granulomatosis with polyangiitis (Wegener disease), or chronic granulomatous infections such as tuberculosis, fungal infections, or histoplasmosis. In idiopathic granulomatous mastitis (IGM), no underlying etiology is identifiable. IGM occurs more frequently in women of childbearing age with elevated hormonal states, such as contraceptive medication and lactation. The most common clinical presentation is a tender retro-areolar mass. Nipple retraction, erythema, swelling, axillary lymphadenopathy may be present [1].

Imaging features of IGM are often nonspecific. The most common mammographic finding is a focal or regional asymmetric density, with potential architectural distortion or skin thickening. MRI shows heterogeneous non-mass-like enhancement. Sometimes, IGM may manifest with abscess formation [1]. An abscess presents as an anechoic or heterogeneously hypoechoic collection with well- or ill-defined margins on ultrasound. Other ultrasound features include heterogeneous breast parenchyma, skin thickening, and edema in the surrounding tissue. The mammographic appearance varies from a well-circumscribed to an ill-defined or irregular opacity. MRI demonstrates a contrast-enhancing rim, central high signal intensity on T2-WI and with diffusion restriction. Most IGM patients with abscess formation are unilateral. However, a few cases of bilateral IGM abscesses have been reported.

On imaging, IGM abscess formation may mimic an invasive breast carcinoma with central necrosis or abscesses in SGM or non-granulomatous infection [1].

The initial treatment of IGM is abscess drainage and immune modulation with a corticosteroid regimen. A methotrexate regimen or surgery may be considered in refractory cases [1].
